# Cognitive Control After ACL Reconstruction: A Cross-Sectional Study on Impaired Proactive Inhibition Compared to Healthy Controls

**DOI:** 10.3390/brainsci15050497

**Published:** 2025-05-12

**Authors:** Jesús Jiménez-Martínez, Alejandro Gutiérrez-Capote, Iker Madinabeitia, David Cárdenas, Francisco Alarcón

**Affiliations:** 1 Faculty of Sports Science, Department of Physical Education and Sport, University of Granada, 18071 Granada, Spain; j.jimenez@ugr.es (J.J.-M.); agcapote@ugr.es (A.G.-C.); 2 Sport and Health University Research Institute (iMUDS), 18007 Granada, Spain; 3 Department of General and Specific Didactics, Faculty of Education, University of Alicante, 03690 Alicante, Spain; iker.madi@ua.es (I.M.); f.alarcon@gcloud.ua.es (F.A.)

**Keywords:** executive function, ACL injury, rehabilitation, proactive inhibition

## Abstract

**Background/Objectives**: Anterior cruciate ligament (ACL) injury is common in interaction sports and has severe physical and psychological consequences. Recent research suggests that neurocognitive factors, such as proactive inhibitory control, may influence injury risk. The present work compares the proactive inhibitory performance ability of athletes with no ACL injury and ACL-rehabilitated athletes (ACLR). **Methods**: This study involved 60 athletes from interaction sports (30 with no history of ACL injury and 30 ACL rehabilitated athletes). During the experimental session, participants performed an executive go–no-go task to assess proactive inhibitory control. **Results**: The ACLR group exhibited higher adjusted-precision response times (*p* = 0.011), higher inhibitory failures response times (*p* < 0.001), poorer accuracy (*p* = 0.003), and higher commission error rate (*p* = 0.026) than the group of athletes with no history of ACL injury. **Conclusions**: Athletes rehabilitated from an ACL injury show inferior performance in proactive inhibitory control, evidenced by lower accuracy and higher reaction times than athletes without a history of injury. Consequently, physiotherapists and exercise professionals should consider cognition during ACL injury rehabilitation and physical retraining before returning to sporting activity.

## 1. Introduction

Anterior cruciate ligament (ACL) injury severely affects athletes’ careers, with a high risk of recurrence and osteoarthritis in the knee [1]. In addition, it negatively impacts mental health, hindering recovery and return to competitive levels [2]. Despite the large economic investments in sports injury detection and prevention, ACL injury is so prevalent that its cost in the United States reaches $3 billion annually [3]. Moreover, ACL injury occurs most frequently in interaction sports such as football or basketball, and in the female population [4].

Despite the enormous interest in understanding the mechanisms that trigger ACL injury, identifying the main factors that may contribute to its occurrence, among the many existing ones, continues to generate controversy. The correct way to understand the injury is through a complex approach that includes factors traditionally studied but of a diverse nature, such as biomechanical, anatomical, neuromuscular, or genetic [5]. However, as the incidence of ACL injury has not decreased in recent years, it has been considered that a neurocognitive perspective could contribute to the understanding of this serious injury [6]. It has to be considered that ACL injury occurs mainly when playing interaction sports, where athletes have to make quick decisions in environments of high variability and uncertainty [7]. This requires rapid processing of sensory stimuli, which increases cognitive demands. In addition, changes of direction and single-leg landings [8] performed under high cognitive demand appear to enhance mechanisms associated with ACL injury, such as increased ground reaction forces or dynamic knee valgus [9]. Recent evidence highlights the benefits of dual-task training, which combines cognitive and physical tasks simultaneously, showing greater improvements in cognitive and physical performance compared to single-task approaches. This has been demonstrated both in individuals with long COVID [10] and in those with knee pain, including ACL injuries [11], supporting the relevance to integrate dual-task strategies or elements of uncertainty into injury risk assessments and preventive training, in order to better detect athletes vulnerable to ACL injuries and contribute to lowering their occurrence [9].

Given the influence of increased cognitive demands on ACL injury risk, cognitive control emerges as a highly relevant variable. The cognitive demands underlying the practice of interaction sports require control over sports gestures to adjust motor responses [12]. Cognitive control regulates and directs behavior toward goals in dynamic and uncertain environments [13]. This regulatory function relies heavily on executive functions (EFs), which are high-level cognitive capacities that enable individuals to plan, adjust, and monitor their actions effectively [14,15]. EFs are considered the underlying mechanisms that make cognitive control possible. According to Diamond [16], three core EFs play a fundamental role in this process: inhibitory control (IC), working memory (WM), and cognitive flexibility (CF). IC refers to the ability to suppress automatic, inappropriate, or impulsive responses while maintaining focused attention amid distractions or shifting circumstances. This skill is essential for avoiding maladaptive behaviors and facilitating goal-directed adaptation. WM allows for the active maintenance and continuous updating of relevant goals, intentions, and information during ongoing tasks, which is particularly critical in performance-driven contexts such as sports. CF, on the other hand, enables individuals to quickly shift strategies or perspectives in response to changing demands, promoting adaptive thinking and behavior in complex and dynamic environments. In the sports context, one of the most important functions is inhibitory control, defined as the ability to regulate or suppress automatic responses and impulses, preventing the execution of inappropriate actions [17], which allows one or more unwanted responses to be withheld while an alternative response is implemented [18]. Several studies have shown that elite athletes exhibit a greater capacity for motor response inhibition than non-athletes [19]. Specifically, an inhibitory response refers to the observable motor or behavioral outcome resulting from the activation of inhibitory control mechanisms, such as successfully suppressing a prepotent movement. A greater capacity for motor response inhibition has also been found in athletes in open sports relative to those in closed-skill sports [20,21]. Practicing in more rapidly changing and unpredictable environments that characterise open sports requires athletes to frequently resolve automatic response control conflicts to optimally adapt to game circumstances [20,21]. A relationship has also been found between response inhibition ability and proactive motor interference. Proactive interference manifests when automatisms hinder the execution of new information [22]. This overlap can induce unwanted decrements in performance [23]. Response inhibition plays a critical role in overcoming interference in contexts involving motor components. Thus, individuals with lower inhibition capacity experience greater difficulties in modifying established skills or show a tendency to accept suboptimal performance [24].

There also appears to be a relationship between an athlete’s neurocognitive ability and a lower risk of musculoskeletal injury [25]. Furthermore, Giesche et al. [26] reported that athletes with poor neurocognitive performance, especially in inhibitory control, cognitive flexibility, and short-term working memory, have kinematic and kinetic patterns of the knee associated with an increased risk of ACL injury. These findings are consistent with a systematic review that identified significant relationships between cognitive performance and motor control, pointing to a common directionality: lower cognitive performance is associated with a higher injury risk profile during movements requiring a high degree of cognitive demand [27]. Along the same lines, it has been observed that in non-contact ACL injuries, there is an impairment in inhibitory control during the execution of motor actions in football players [28]. Therefore, inhibition may play a key role in preventing ACL injuries, as the processing and selection of a large amount of information within a limited amount of time may affect both the quality of the decision made and the motor response [29].

On the other hand, physical activities dominated by overt motor skills involve outcome predictions about the actions of opponents and teammates to produce fast and accurate responses [30,31]. To do so, the athlete needs to use proactive cognitive control, a form of top-down regulation characterised by early selection processes that optimally bias attention, perception, and action systems in a goal-driven manner [32]. Proactive inhibitory control specifically refers to the anticipatory suppression of prepotent motor responses before a triggering event occurs, preparing the motor system to withhold or delay action if needed [33,34]. Proactive cognitive control represents a part of inhibitory control, which would consist of two systems in the dual model mechanics framework [32]. Neurophysiologically, proactive inhibition is supported by increased activity in the dorsolateral prefrontal cortex (DLPFC) and its interactions with the motor cortex and basal ganglia, which facilitate goal-oriented motor regulation [35]. In the context of open-skill sports where athletes constantly adapt to unpredictable scenarios, proactive inhibitory control plays a crucial role in avoiding premature or inappropriate motor responses, such as withholding a sprint start to avoid offside or pausing a swing until the opponent’s movement unfolds [32,33,35]. Some studies have observed that elite athletes possess greater proactive cognitive control [31] and a better capacity for proactive inhibition [36], which in disciplines such as interaction sports may be key to sports performance, as athletes must adjust their proactive control according to environmental cues, for example, by modifying their response depending on the opponent’s postures when attacking or defending. In this regard, previous studies have highlighted general cognitive deficits associated with ACL injuries, such as slower processing speed and impaired reaction time [37], but few have isolated specific executive function components critical for motor planning and injury prevention. Furthermore, while studies like Emami et al. [38] have examined the impact of various cognitive tasks on postural control tasks in ACL-injured patients, no study has been conducted to explore and explain possible relationships between proactive inhibitory control in groups with and without ACL injury.

Therefore, assessing proactive inhibitory control can provide information on how athletes interact in real sports environments and their risk of ACL injury. Although some studies have assessed cognitive performance in athletes while performing tasks involving decision-making [39,40], no studies have assessed proactive inhibitory control in athletes with a history of ACL injury. Therefore, the present work aims to compare the proactive inhibitory performance ability of athletes with no ACL injury and ACL-rehabilitated athletes (ACLR). This study will help physical trainers and readaptators understand the importance of cognitive control over ACL injury and help them become aware of the importance of considering this variable in their ACL injury detection and retraining programmes.

## 2. Materials and Methods

### 2.1. Type of Study

The present work was designed as a cross-sectional study aimed at comparing the proactive inhibitory performance ability between athletes with no anterior cruciate ligament (ACL) injury and ACL-rehabilitated athletes (ACLR).

### 2.2. Participants

Herein, 60 participants were recruited and divided into two groups: 30 athletes with no history of ACL injury and 30 who had completed rehabilitation of an ACL injury. Data were collected regarding age, weight, height, and the sport modality practiced by each participant. All participants played open-modality sports. Demographic details of the participants are presented in Table 1. This study was conducted by the Declaration of Helsinki and approved by the university’s institutional review board (approval number: 3110/CEIH/2022) on 30 December 2022, in Granada.

In order for participants to be selected, they had to meet the following inclusion criteria: (1) young adults aged 18–35 years, (2) no diagnosis of psychiatric or neurological disorders (such as ADHD and depression), (3) no history of psychiatric or neurological injury (such as ADHD and depression), (4) no history of psychiatric or neurological disorders, (5) no history of severe brain injury or traumatic brain injury, (6) no diagnosis of cardiovascular and/or metabolic diseases, (7) normal or corrected vision, and (8) active athletes in interaction sports with at least two sport-specific training sessions per week and at least 3 years of continuous federated sports experience. On the other hand, a selection criterion was established for each group assignment. For the group of athletes rehabilitated from the ACL injury, they were required to be medically cleared, or at least one year had passed since the ACL reconstruction. For the other group, participants could not have a history of ACL injury.

### 2.3. Procedure

This study commenced following its approval by the Ethics Committee of the University of Granada. Participant recruitment took place from February to April 2023. The sample size was determined with reference to previous studies employing similar study designs, ensuring methodological consistency and adequate statistical power [41]. Data collection was conducted between May and July 2023, and the data were subsequently processed and analysed during August and September 2023. Manuscript writing began in October 2024 and continued until the final version was completed in April 2025, when this study was submitted for publication.

#### 2.3.1. Familiarisation Session

During the familiarisation session, participants were given a detailed explanation of this study and precise instructions about the cognitive test they would perform during the experimental session. In addition, participants signed an informed consent form to participate. Finally, they had the opportunity to practice the go–no-go cognitive test until they were sure that they fully understood it. This session lasted approximately one hour.

#### 2.3.2. Experimental Session

The experimental session took place in one of the laboratories at the Faculty of Physical Activity and Sport Sciences of the University of Granada. This space was quiet and free of noise, which allowed the session to be conducted without interruptions or distractions. Participants were asked to avoid intense exercise in the 48 h prior to the experimental session [42], to fast for the four hours preceding the session, to ensure at least seven hours of sleep the night before [43], to refrain from consuming alcohol in the previous 24 h [44], and to avoid ingesting caffeine or theine in the 12 h before the session [45]. The duration of the experimental session was approximately 2 h.

#### 2.3.3. Cognitive Task

**Figure 1:**
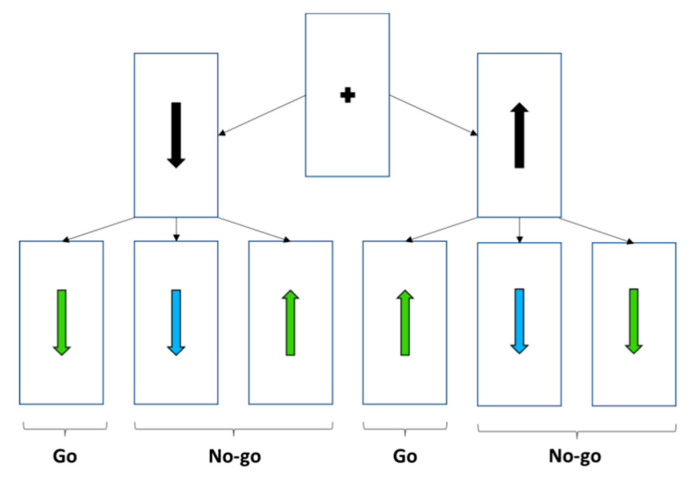
Task rule linked to the interaction between colour and direction of the pre-stimulus and target stimulus. Each trial began with the presentation of a black arrow (pointing either upward or downward) displayed for 500 ms. After a variable delay of 1000 to 2000 ms, a coloured arrow appeared. In go trials, the arrow turned green and maintained the same orientation as initially. In no-go trials, the arrow either turned green but changed orientation or turned turquoise. Participants were instructed to press the button as quickly as possible only when the arrow turned green and maintained its original orientation. Responses were to be withheld in all other cases.

During the experimental session, participants performed an executive go–no-go task to assess inhibitory control. A modified version of the Vocat et al. [46] study was programmed in SuperLab 6.3.0 software. Each trial started with a black arrow (vertical or inverted) in the centre of the screen, which remained visible for a variable time between 1000, 1500, and 2000 ms to prevent the participants from systematically anticipating the change in colour or orientation of the arrow. This black arrow was replaced by a coloured arrow (green or turquoise), which could maintain the same orientation or be inverted (180°). The coloured arrow remained on the screen until the subject’s response on go trials or for a maximum of 1500 ms on no-go trials. Between each trial, there was a time interval in which a blank screen appeared for 500 ms, followed by a central fixation cross for another 500 ms. Participants had to press the response key as fast as possible if the black arrow turned green and maintained the same orientation (‘go’ trials). In contrast, if the black arrow turned green but changed orientation or turned turquoise regardless of orientation, they were not to press (‘no go’ trials) (see Figure 1). After a correct response, either a press within the time limit on go trials or inhibition on no-go trials, the word ‘correct’ was displayed for 500 ms. In case of an error of commission or omission, the visual feedback displayed ‘incorrect’. During the explanation of the task, the importance of achieving a balance between accuracy and speed of response was highlighted. This balance was directly associated with the ability to predict the target stimulus as a function of probability and available time [47].

**Figure 2:**
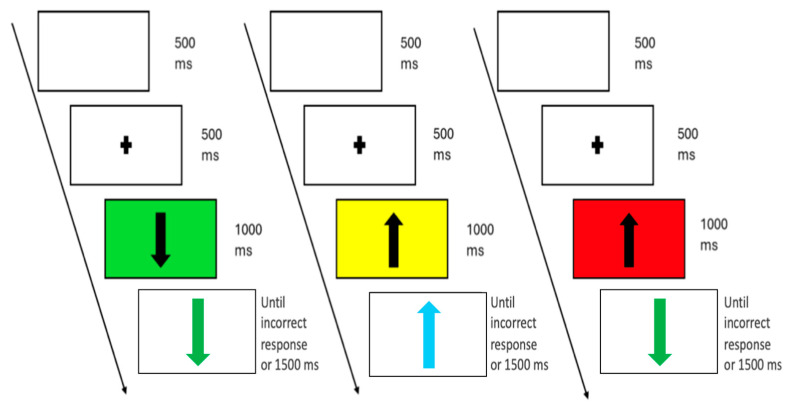
Example of a sequence of 3 trials from the go–no-go task. Each trial began with the presentation of a white display followed by a central fixation cross. Next, a black arrow (pointing upward or downward) appeared over a coloured background (green, yellow, or red). The background colour indicated the probability of facing a go or no-go stimulus (green = low no-go probability (13.3%), yellow = medium no-go probability (33.3%), and red = high no-go probability 53.3%). In go trials (66.67%), the arrow turned green and maintained its original orientation, prompting participants to press the response key as quickly as possible. In no-go trials, participants were instructed to withhold their response if (i) the arrow turned green but changed its orientation, or (ii) the arrow turned turquoise, regardless of orientation. The figure shows examples of a go trial following a green background, a no-go trial due to a turquoise arrow after a yellow background, and a no-go trial due to an orientation change following a red background.

A visual cueing procedure prepared the participant for the appropriate target stimulus and enhanced the proactive preparatory phase [48,49]. Specifically, the target stimuli were always preceded by a background colour that could be one of three possible colours (green, yellow, and red), appearing next to the black arrow, indicating three degrees of probability (high, medium, and equally likely) that a go trial or a no-go trial would follow [36]. The green background indicated a 13.33% probability of an incongruent (no-go) stimulus, with 52 go trials and 8 no-go trials. The yellow background corresponded to a 33.33% probability of occurrence of the incongruent stimulus, with 40 go and 20 no-go trials. Finally, the red background reflected a 53.3% probability of an incongruent stimulus, with 28 go and 32 no-go trials. The order of the presentation of trials for each background colour was randomised entirely (see Figure 2). Participants were not informed of the probability assigned to each pre-index type.

**Figure 3:**
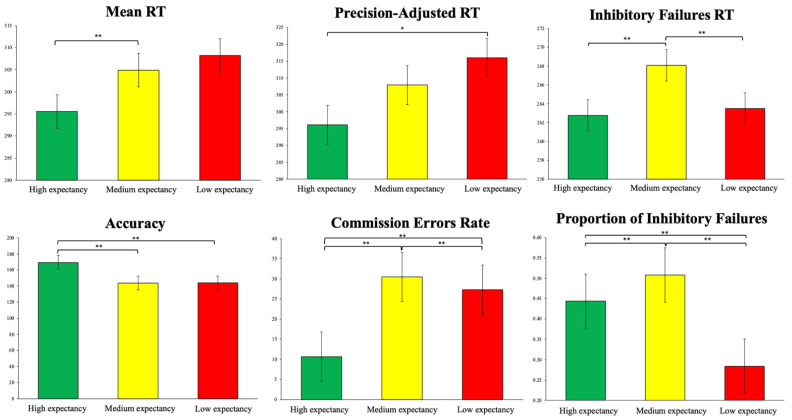
Behavioral performance for each expectancy condition of the go–no-go task, with mean ± standard error. * Indicates *p* < 0.01; ** indicates *p* < 0.001. A mixed-effects analysis was conducted to verify these results.

The cognitive test was organised into 3 blocks with 180 trials, of which 120 were go and 60 were no-go (see Figure 3). Before each block, a calibration block comprising 14 trials (10 go and 4 no-go) was performed to calculate the response time available for each go trial in the task. This set an individualised response time limit, adjusted to 90% of the average response time obtained in the calibration block, which meant that participants had to respond at least 10% faster [46]. A 2-min rest period was included between each block of the task.

The experiment consisted of 540 trials, with 360 go and 180 no-go. In addition, there were 42 additional trials in each of the three calibration blocks. Thus, the task comprised 2/3 go trials (66.67%) and 1/3 no-go trials (33.3%). The total duration of the task was 1 h. The go–no-go trial paradigm allowed for the measurement of participants’ proactive inhibition capacity, as it favours preparatory processes for the inhibition of motor behaviour prior to the occurrence of an imperative stimulus. This reflects active maintenance of task goals, facilitating cognitive control and response regulation [33,50]. On the other hand, several indicators were used to assess cognitive test performance: number of hits (key press within or outside the time limit on go trials and response inhibition on no-go trials), the number of errors of commission (key press on no-go trials), and the number of errors of omission (no key press on go trials or key press before the appearance of the target stimulus) [33]. We also measured the mean response time (TRm) in milliseconds and the accuracy-adjusted TRm on correct trials.

#### 2.3.4. Statistical Analysis

The statistical analysis of this study was conducted in two main phases. First, to determine whether participants associated the frequency of the “go” stimulus with specific background colours—such as attributing a higher likelihood of needing to press the button to the green background—behavioral performance variables from the cognitive task (i.e., response time adjusted for accuracy, commission errors, and omission errors) were compared across the three expectancy grades. This comparison was performed using mixed-effects modeling: the dependent variable in each model was the respective performance indicator; the fixed effect was the expectancy condition, while participant was included as a random effect with random intercepts to account for inter-individual variability (e.g., Commission_errors ~ Expectancy + (1|Participant)).

Second, to assess differences in cognitive performance between experimental groups, independent sample analyses were conducted. Parametric or non-parametric tests were applied depending on the distributional properties of the data, as assessed via the Shapiro–Wilk test.

For each mixed-effects model, a Bonferroni correction was applied to adjust for multiple comparisons among expectancy conditions (i.e., high vs. medium vs. low expectancy). All statistical analyses were performed using RStudio (version 2024.04.2), and the significance threshold was set at *p* < 0.05.

## 3. Results

### 3.1. Verification of the Attribution of Go Stimulus Appearance Percentages to Each Condition of the Cognitive Task

The mixed-effects analysis showed significant results, indicating that participants correctly attributed the expected probability to each condition. In the behavioural performance variables, it was observed that for the high expectancy go condition, the mean RT was significantly lower than in the medium- and low-expectancy go conditions (t = −4.662, *p* < 0.001; t = −4.311, *p* < 0.001, respectively). No differences were found between medium- and low-expectancy go conditions (t = −2.067, *p* = 0.131). For the precision-adjusted RT, the low-expectancy go condition was significantly higher than the high-expectancy go condition (t = 2.844, *p* < 0.01). For the inhibitory failures RT, the low expectancy go condition was significantly higher than the high expectancy go condition (t = 24.262, *p* < 0.001), and the medium expectancy go condition was significantly higher than the high expectancy go condition (t = 124.344, *p* < 0.001). Also, the low expectancy go condition was significantly higher than the medium expectancy go condition (t = 142.652, *p* < 0.001). Regarding accuracy, the high-expectancy go condition showed higher accuracy than the medium- and low-expectancy go conditions (t = 25.519, *p* < 0.001; t = 25.444, *p* < 0.001, respectively). No differences were found between the medium- and low-expectancy go conditions (t = −0.074, *p* = 1.000). For the commission error rate, the low-expectancy go condition was significantly higher than the high-expectancy go condition (t = 11.950, *p* < 0.001), and the medium-expectancy go condition was significantly higher than the high-expectancy go condition (t = 14.347, *p* < 0.001). Regarding the proportion of inhibitory failures, the low-expectancy go condition was significantly higher than the high-expectancy go condition (t = 7.353, *p* < 0.001), and the medium-expectancy go condition was significantly higher than the high-expectancy go condition (t = 4.846, *p* < 0.001). Also, the low-expectancy go condition was significantly higher than the medium-expectancy go condition (t = 10.481, *p* < 0.001). These results are visually presented in Figure 3.

### 3.2. Cognitive Performance Differences Between Experimental Groups

Regarding behavioural variables for the entire task, it was observed that the ACLR group had significantly longer mean RT (t = −3.327, *p* = 0.002), precision-adjusted RT (U = 134; *p* = 0.011), and inhibitory failures RT (t = −4.828, *p* < 0.001) compared to the healthy group. Regarding accuracy, the ACLR group showed lower accuracy than the healthy group (t = 3.162, *p* = 0.003). Additionally, the ACLR group had a significantly higher commission error rate (t = −2.3082; *p* = 0.026) and proportion of inhibitory failures (t = −2.764, *p* = 0.008) than the healthy group. For comparison of cognitive performance between groups for each expectancy go condition, please see Table 2.

The healthy group outperformed the ACLR group in the task variables across all expectancies of go conditions, with the most pronounced differences in the high and medium expectancies of go conditions. The ACLR group exhibited higher response times and poorer accuracy. These findings suggest that ACLR individuals may experience processing speed and response inhibition deficits, with performance varying depending on task difficulty.

These results are visually presented in Table 2.

## 4. Discussion

The present study aimed to compare the performance in proactive inhibitory control between athletes without an ACL injury and those rehabilitated after an ACL injury. After analysing the results, it was found that healthy athletes had better performance in proactive inhibitory control, represented by better accuracy and lower response times compared to rehabilitated athletes. In conclusion, the rehabilitated athletes had worse cognitive performance despite completing the recovery process.

Athletes rehabilitated from an ACL injury had worse behavioural performance on the task, as they had lower hits and longer reaction times than healthy athletes. Several authors have reported worse cognitive performance in dimensions such as reaction time or processing speed in injured athletes and athletes at risk for ACL injury [51]. However, studies have also found no negative effect on reaction time and other dimensions of cognition [37]. This suggests that ACL injury is not directly related to all dimensions of cognition, but rather to specific ones that seem to influence the mechanisms responsible for the injury [52]. However, since the study design is cross-sectional, it is impossible to establish a cause-and-effect relationship, and the direction of the relationship remains uncertain. It is known that athletes with poorer cognitive performance have a higher risk of musculoskeletal injury [25], but the fact that the group of ACL rehabilitees have poorer behavioural performance could be because poor cognitive performance was present before the injury and contributed to its occurrence, or, conversely, that the injury itself influenced the deterioration of cognitive performance.

On the other hand, in the present study, athletes rehabilitated from ACL injury presented differences in the performance of the three conditions according to probabilism. Adapting to probabilistic contexts allows for the generation of more accurate expectations in the task, adapting behaviour according to the context [53]. Previous studies have mainly assessed the effect of ACL injury on basic cognitive skills (reaction time, visual attention, or processing speed), but executive functions involved in the performance of the aforementioned skills have not been taken into account. This makes it difficult to contrast the results of this study with previous findings. Some studies have included cognitive tests that assess executive functions, but they have been applied in dual-task contexts (tasks with concomitant motor and cognitive demands). It should be noted that in these studies, a worse motor performance of athletes has been observed due to the dual task, enhancing the mechanisms linked to the risk of ACL injury [9]. In line with these results, it has been observed that poorer cognitive performance is associated with a higher risk of injury in game contexts, where cognitive demands are high [27]. Consequently, it is important to develop reasonable cognitive control in athletes to prevent ACL injuries.

The results of the present study indicate that ACL-rehabilitated athletes are less effective in using proactive inhibitory control compared to their healthy peers, which may limit their ability to respond quickly and effectively in situations requiring decision-making [54]. These circumstances favour ACL injury, as increased cognitive demands enhance ACL injury mechanisms such as increased ground reaction forces (GRF) or knee abduction [9,55,56]. Consequently, the athlete must respond adaptively by adjusting available resources and prioritising the processing of the most relevant information to resolve conflicts [57]. This decrease in inhibition could also affect the ability to provide a more adaptive motor response to small changes in the environment. Inhibition is important for controlling interference [17,58]. It is often necessary to suppress unwanted action tendencies to overcome interference and successfully execute the target task [59]. Proactive cognitive control would facilitate inhibition or adaptation of future actions, which ensures greater precision in motor execution, adjusting to the demands of the environment. In this sense, poorer performance on inhibitory control could explain the lack of capacity of the system to dampen ground impact forces, altering the dynamics of the individual and increasing the risk of ACL injury [28]. The mechanisms of information transmission between brain structures and peripheral sensory receptors are key to reacting to environmental stimuli efficiently. Reduced connectivity between the left sensory cortex and the right cerebellum, essential for motor coordination, has been found to increase the risk of ACL injury [60]. The sum of reduced brain connectivity and minor mental errors in coordination and movement planning may limit the rapid transmission of proprioceptive information, hindering the muscle contractions needed to dampen impact forces at the joint [61]. Future studies could investigate how to improve neurocognitive performance in ACL prevention and rehabilitation programmes.

The present work provides a further step in studying the relationship between cognition and ACL injury, focusing mainly on how proactive inhibitory control may be affected by ACL injury. Furthermore, this study highlights that cognitive performance is a relevant variable in the context of sports injuries and should be considered in ACL injury prevention and rehabilitation programmes. However, this study has certain limitations that need to be considered. Firstly, it should be noted that this study has a cross-sectional design, and it is not possible to obtain a cause-and-effect relationship. Secondly, although the sample size was based on previous studies with similar designs, a larger cohort would provide greater statistical power and the generalizability of the findings. Therefore, future studies could analyse the evolution of cognitive performance throughout the different recovery phases. To this end, three measurements could be taken: during the preoperative period, in the postoperative period, and at the end of rehabilitation. Additionally, future research could optimise the dual-task protocol by increasing its complexity or tailoring it to the specific stages of recovery, thereby enabling a more precise assessment of progressive cognitive-motor adaptations. Furthermore, implementing extended follow-up periods would facilitate the investigation of the long-term effects of rehabilitation on both cognitive and motor functions, offering a more comprehensive understanding of recovery dynamics. This would provide a complete picture of the relationship between neurocognitive factors and ACL injury.

## 5. Conclusions

Athletes rehabilitated from an ACL injury show inferior performance in proactive inhibitory control, as evidenced by lower accuracy and higher reaction times compared to athletes without a history of injury. Consequently, trainers and exercise professionals should consider cognition during both ACL injury rehabilitation and physical retraining prior to returning to sporting activity.

## Figures and Tables

**Table 1:** Descriptive statistics of the sample.

	Healthy Athletes	ACLR Athletes
N (Male/Female)	20/10	22/8
Years of practical experience	9.26 ± 1.44	8.83 ± 1.21
Age (years)	21.24 ± 5.87	22.36 ± 6.12
Height (m)	179.11 ± 15.32	80.66 ± 13.65
Body mass (kg)	175.93 ± 9.40	76.34 ± 9.65
BMI (kg·m^−2^)	20.23 ± 0.43	20.93 ± 0.34

Data values are expressed as mean ± standard deviation. N, sample size; m, meters; kg, kilograms; BMI, body mass index.

**Table 2:** Comparison of cognitive performance in the go–no-go task between athletes with and without a history of ACL injury.

	Healthy Athletes		ACLR Athletes		
	M	SD	M	SD	*p*
Overall task					
Mean RT	284.73	31.20	325.22	57.31	**0.002**
Precision-Adjusted RT	288.27	29.69	328.28	56.02	**0.011**
Inhibitory Failures RT	238.81	30.31	297.24	54.9	**<0.001**
Accuracy	471.43	41.22	439.46	31.15	**0.003**
Commission Errors Rate	58.28	32.11	80.44	25.85	**0.026**
Proportion of Inhibitory Failures	0.33	0.18	0.45	0.15	**0.008**
High-expectancy condition					
Mean RT	272.41	37.61	324.72	56.97	**<0.001**
Precision-Adjusted RT	272.51	35.49	325.05	56.13	**<0.001**
Inhibitory Failures RT	236.87	30.28	294.52	55.06	**<0.001**
Accuracy	170.57	6.20	167.59	8.63	0.181
Commission Errors Rate	8.90	5.60	12.8	5.52	**0.011**
Proportion of Inhibitory Failures	0.37	0.23	0.55	0.23	**0.011**
Medium-expectancy condition					
Mean RT	287.67	31.59	326.50	59.13	**0.002**
Precision-Adjusted RT	290.73	28.42	328.77	57.00	**<0.001**
Inhibitory Failures RT	242.03	30.34	300.04	55.36	**<0.001**
Accuracy	151.50	17.81	132.82	14.84	0.068
Commission Errors Rate	24.76	14.18	37.12	13.55	**0.002**
Proportion of Inhibitory Failures	0.42	0.23	0.65	0.21	**0.002**
Low-expectancy condition					
Mean RT	295.18	27.87	325.06	59.49	**0.002**
Precision-Adjusted RT	301.60	27.89	333.10	56.81	**0.018**
Inhibitory Failures RT	237.54	30.31	295.35	55.22	**0.014**
Accuracy	149.37	17.80	136.09	10.15	**0.010**
Commission Errors Rate	24.72	12.76	30.2	8.49	0.074
Proportion of Inhibitory Failures	0.26	0.13	0.32	0.08	0.093

Results for *p* < 0.05 are highlighted in bold. Abbreviations. ACLR, ACL-rehabilitated; RT, response time; SD, standard deviation.

## Data Availability

The data from our study have been made publicly available in an open-access repository. The original data presented in this study are openly available, and the dataset can be accessed at the following link: https://osf.io/bwgkn/files/osfstorage/67fcd3ff922105c580763bcb (accessed on 13 April 2025).
